# Electroconvulsive stimulation attenuates chronic neuroinflammation

**DOI:** 10.1172/jci.insight.137028

**Published:** 2020-09-03

**Authors:** Smadar Goldfarb, Nina Fainstein, Tamir Ben-Hur

**Affiliations:** Department of Neurology, The Agnes Ginges Center for Human Neurogenetics, Hadassah – Hebrew University Medical Center, Jerusalem, Israel

**Keywords:** Inflammation, Neuroscience, Multiple sclerosis

## Abstract

Electroconvulsive therapy is highly effective in resistant depression by unknown mechanisms. Microglial toxicity was suggested to mediate depression and plays key roles in neuroinflammatory and degenerative diseases, where there is critical shortage in therapies. We examined the effects of electroconvulsive seizures (ECS) on chronic neuroinflammation and microglial neurotoxicity. Electric brain stimulation inducing full tonic-clonic seizures during chronic relapsing–progressive experimental autoimmune encephalomyelitis (EAE) reduced spinal immune cell infiltration, reduced myelin and axonal loss, and prevented clinical deterioration. Using the transfer EAE model, we examined the effect of ECS on systemic immune response in donor mice versus ECS effect on CNS innate immune activity in recipient mice. ECS did not affect encephalitogenicity of systemic T cells, but it targeted the CNS directly to inhibit T cell–induced neuroinflammation. In vivo and ex vivo assays indicated that ECS suppressed microglial neurotoxicity by reducing inducible NOS expression, nitric oxide, and reactive oxygen species (ROS) production, and by reducing CNS oxidative stress. Microglia from ECS-treated EAE mice expressed less T cell stimulatory and chemoattractant factors. Our findings indicate that electroconvulsive therapy targets the CNS innate immune system to reduce neuroinflammation by attenuating microglial neurotoxicity. These findings signify a potentially novel therapeutic approach for chronic neuroinflammatory, neuropsychiatric, and neurodegenerative diseases.

## Abstract 2

Electroconvulsive stimulation reduces neuroinflammation by attenuating microglial neurotoxicity in a clinically relevant model for chronic multiple sclerosis.

## Introduction

Neuroinflammation is considered a major mediator of brain injury in multiple chronic neurological and neuropsychiatric disorders, and it presents an important therapeutic target for intervention (1). The brain’s innate immune system, represented mainly by microglia, plays a leading role in chronic neuroinflammation–induced brain injury (2). Toxic microglial activation occurs both when the disease is inflicted by an autoimmune process or secondary to pathologic cascade of events triggered by misfolded proteins in neurodegenerative diseases (3, 4). Activated microglia take on a proinflammatory phenotype, producing inflammatory cytokines, chemokines, and free radicals, such as nitric oxide (NO) and reactive oxygen species (ROS) (5, 6). The confinement of the injurious neuroinflammatory disease process to the CNS — which is dominated by innate toxic microglia, occurring behind the blood-brain barrier (BBB) — creates a major challenge for developing targeted and effective therapies. This is exemplified in chronic-progressive multiple sclerosis (MS), in which the BBB is significantly less permeable (7), and immune pathogenesis is compartmentalized within the CNS (8–10). Microglia play a key role in the inflammatory damage in chronic MS (11). Indeed, current MS therapies mainly targeting the peripheral adaptive immune system are effective during the relapsing phase but fail in progressive MS (12). The lack of therapies for chronic MS emphasizes the unmet need for developing treatments that target the CNS directly (13), modulating its toxic innate immune system.

Electroconvulsive therapy (ECT) is a CNS-targeted treatment that is highly effective in psychiatric disorders such as major depression and catatonic schizophrenia (14). Suggested mechanisms by which ECT affects the brain include increased hippocampal neurogenesis (15) and gliogenesis (16), elevation of trophic factors (BDNF, NGF, VEGF, FGF) (17–20), and modulation of various neurotransmitters (21). ECT may downregulate immune activation (22) by inhibiting its markers of cellular activation (23), microgliosis (24, 25), altering TNF-α (26) and IL-6 (27) levels. The therapeutic implications of these effects are unknown. Since innate immunopathogenic mechanisms may also underlie depression (28, 29), which is most responsive to ECT, we examined the effects of ECT on neuroinflammation.

Here, we examined the therapeutic effects of ECT on chronic neuroinflammation in experimental autoimmune encephalomyelitis (EAE), the animal model of MS. Repeated electroconvulsive seizures (ECS) arrested clinical deterioration in chronic EAE, decreased neuroinflammation, and reduced demyelination and axonal loss. Use of the transfer EAE model showed that ECS affects the CNS directly, not attenuating systemic adaptive immune encephalitogenicity. ECS’s therapeutic effect was mediated by reducing microglial toxicity, without transforming their phenotype. We conclude that electric brain stimulation attenuates chronic neuroinflammation–related CNS injury by reducing microglial toxicity.

## Results

### ECS markedly attenuates chronic neuroinflammation in chronic-progressive Biozzi EAE.

In order to achieve effective ECS, we established the electric threshold to cause a full tonic-clonic seizure, observed as static limb extension, followed by tonic-clonic limb and tail movements for 10–20 seconds. After seizures, mice appeared to be postictal (conscious but not moving willingly) for about 2 minutes. The electric threshold was 1.2 ± 0.18 mC (*n* = 7) in Biozzi mice and 1.6 ± 0.31 mC (*n* = 4) in SJL/J mice. In further experiments, we treated mice with EAE. We applied ECS with twice the average threshold, similar to common clinical practice in human patients and as published for other ECS protocols (30, 31).

Most EAE models are useful for studying systemic immune pathogenesis but do not recapitulate pathologically and clinically the chronic aspects of MS (32). In the Biozzi mouse EAE model (33), mice undergo an acute relapse–remitting phase, followed by chronic relapsing and progressive disease. This chronic phase, developing usually beyond day 35 after induction, is characterized by microgliosis, astrogliosis, chronic neuroinflammation, demyelinated lesions, remyelination, and progressive axonal loss, which are suggestive of an immunomediated neurodegenerative process (34). These characteristics make the Biozzi EAE model relevant for developing targeted therapies for chronic neuroinflammation (35). We therefore used it to examine the effect of ECS on neuroinflammation.

**Figure 1:**
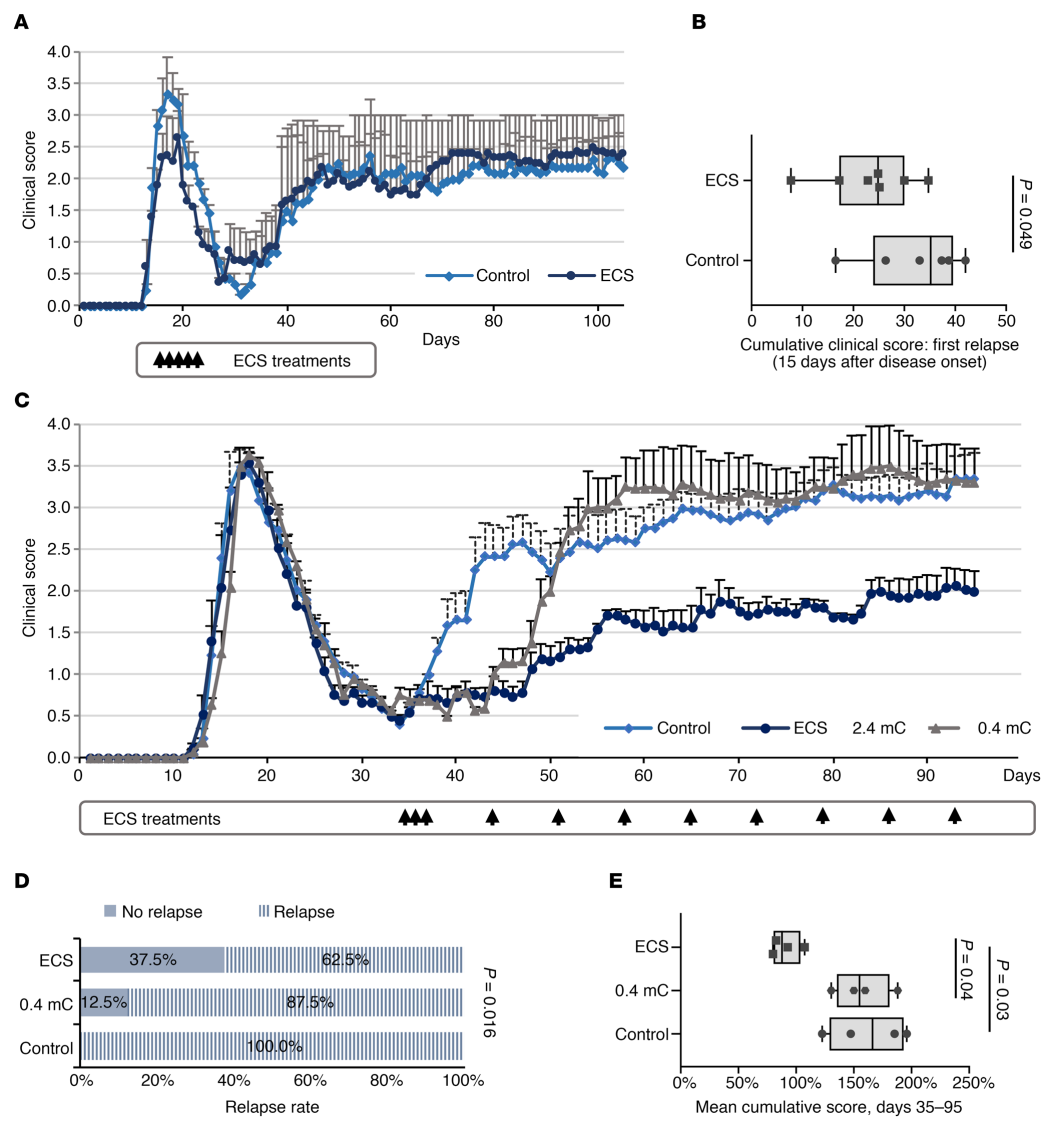
ECS attenuates chronic EAE. (**A** and **B**) ECS attenuates the first EAE relapse. Five ECS (*n* = 7 mice) or sham (control, *n* = 6 mice) treatments were performed on alternating days, starting on the first day of clinical deterioration, individually per each mouse. Average daily clinical scores show mild improvement in the clinical severity of the first relapse of EAE in ECS-treated mice. Data represent mean ± SD (**A**). The cumulative score of the first relapse (lasting in average 15 days) was significantly lower in ECS-treated EAE mice (**B**). Experiment was repeated twice. *P* values calculated with Student’s unpaired *t* test. (**C**–**E**) ECS attenuates chronic EAE. Biozzi EAE mice were treated with ECS (2.4 mC, *n* = 16 mice), a subthreshold current (0.4 mC, *n* = 8 mice), or sham treatment (control, *n* = 17 mice) starting before the second relapse. Treatments were given on 3 consecutive days (35–37 p.i.), followed by weekly maintenance treatments until day 95 p.i. Average daily clinical scores show that ECS (but not subthreshold stimulation) improved the clinical course of chronic EAE (**C**). Data represent mean ± SEM. The experiment was repeated 4 times. ECS significantly reduced the relapse rate as compared with both sham and subthreshold treatment groups. Relapse was determined by an increase of > 1 point in clinical score at any time point between day 35 and 95 p.i. (**D**). *P* values were calculated by 3-way χ^2^ test. ECS significantly reduced the cumulative clinical score of days 35–95 p.i. Dots represent the mean cumulative score of the 4 individual experiments (**E**). *P* value was calculated by using 1-way ANOVA followed by Tukey’s post hoc test. *f*_(2,8)_ = 6.3, *P* = 0.023. Box-and-whisker plots show quartiles with median, and with minima and maxima at the bottom and top whiskers, respectively.

We first treated Biozzi mice with ECS (*n* = 7) or sham treatment (*n* = 6) at clinical presentation of the first relapse. Treatment was initiated on the first day of clinical signs in individual mice (tail drop, presenting postinduction [p.i.] days 13–17), and 4 additional ECS sessions were applied on alternating days. The clinical effect was evaluated by calculating the cumulative score of the acute (first) relapse, typically spanning 15 days. Mild but significant improvement was observed, as evident by reduced burden of disease during the first relapse (Figure 1, A and B). The beneficial effect was transient, as there was no difference in the severity of the progressive phase of EAE (Figure 1A).

**Figure 2:**
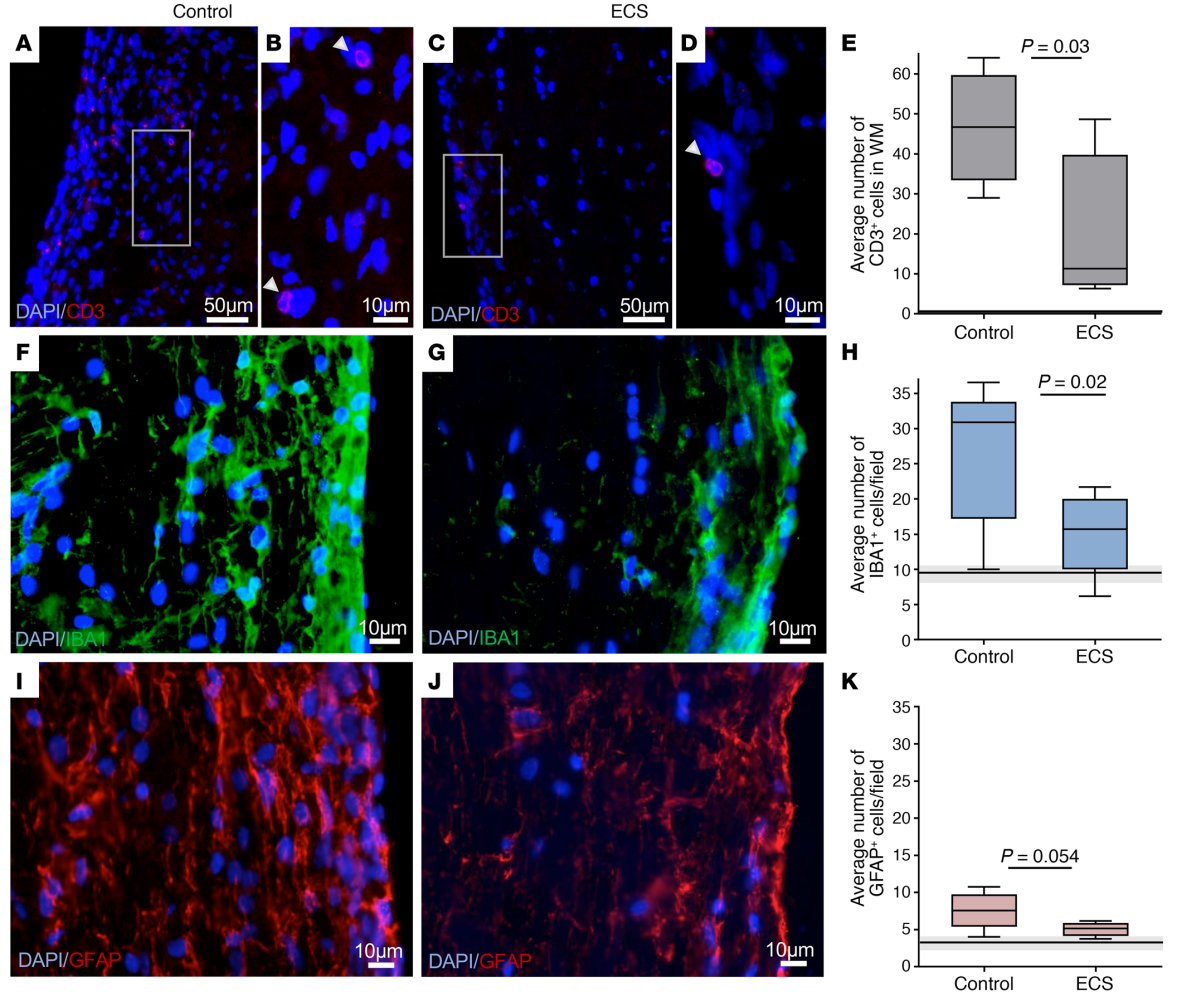
ECS inhibits chronic neuroinflammation. Pathological analysis was obtained at the end of the experiment at day 95 p.i. from ECS-treated versus sham-treated (control) mice as shown on Figure 1C. In all graphs, the horizontal lines (and gray zone) represent the median value (± minima/maxima) of naive, age-matched Biozzi mice. (**A**–**E**) Immunofluorescence staining for CD3 in control EAE (**A** and **B**) and ECS-treated EAE (**C** and **D**) showed that ECS induced a 59% reduction in the total amount of CD3^+^ T cells in spinal cord white matter CD3^+^ cell quantification is provided as total number of cells per (uniformly cut) section (**E**). The white squares in **A** and **C** are enlarged in **B** and **D**, respectively; arrowheads point to CD3^+^ cells. (**F**–**H**) Immunofluorescence staining for IBA1 in control EAE (**F**) and ECS-treated EAE (**G**) showed that ECS induced a 44% reduction in IBA1^+^ cells in spinal cord white matter (SC WM) (**H**). IBA1^+^ cell quantification is provided as number of cells per microscopic field. (**I**–**K**) Immunofluorescence staining for GFAP in control EAE (**I**) and ECS-treated EAE (**J**) showed that ECS induced a 35% reduction in GFAP^+^ cells in SC WM (**H**). GFAP^+^ cell quantification is provided as number of cells per microscopic field. *P* values calculated with Student’s unpaired *t* test. Box-and-whisker plots show quartiles with median, and with minima and maxima at the bottom and top whiskers, respectively.

**Figure 3:**
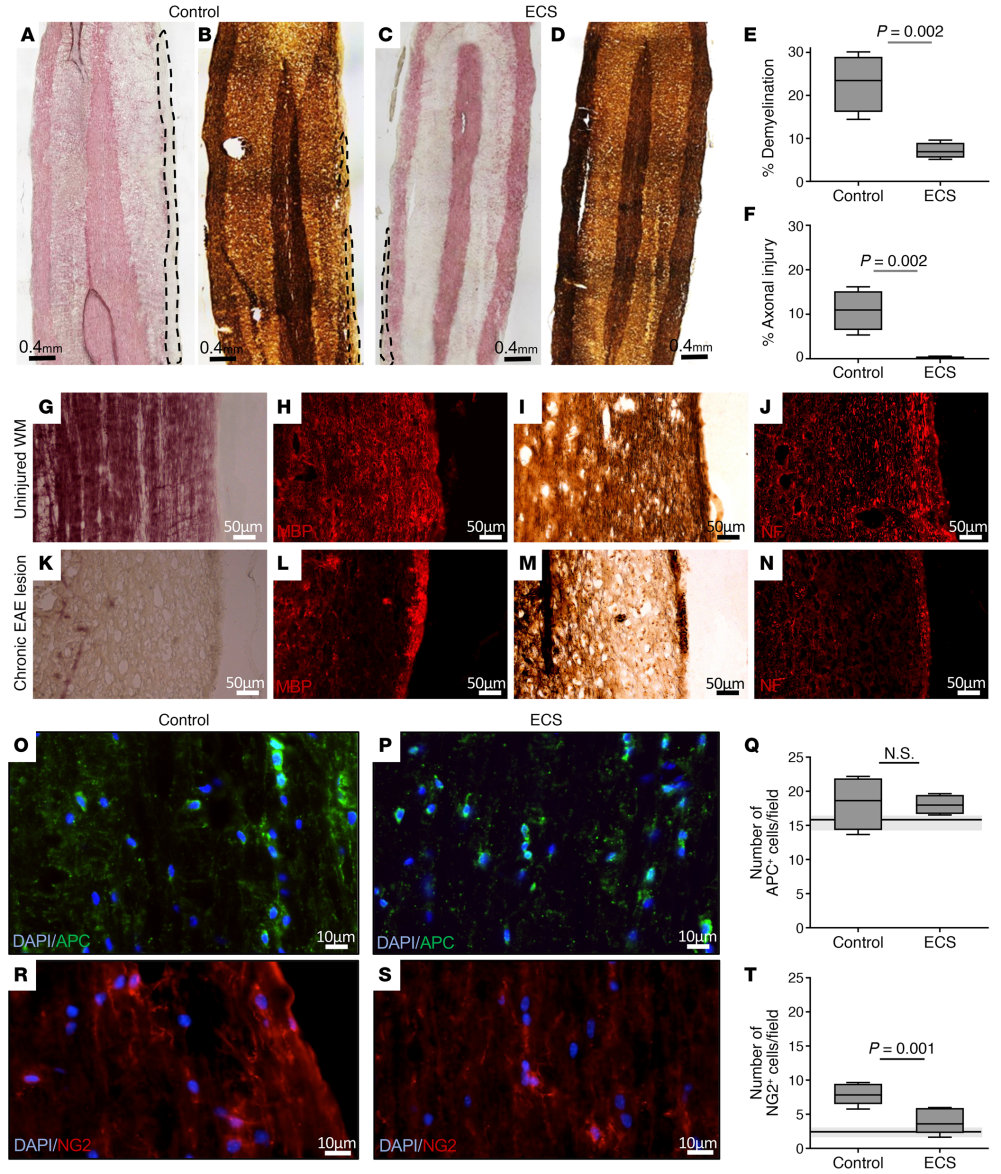
ECS reduces demyelination and axonal injury in chronic EAE. Pathological evaluation was performed at the end of the experiment at day 95 p.i. from ECS-treated versus sham-treated mice as shown on Figure 1C. Horizontal lines (and gray zone) in **Q** and **T** represent median value (± minima/maxima) of naive age-matched Biozzi mice. (**A**–**F**) Gold-Black staining for Myelin (**A** and **C**) and Bielschovsky staining for axons (**B** and **D**) in spinal cords of control EAE (**A** and **B**) and ECS-treated EAE mice (**C** and **D**). Black/white dashed line shows areas of myelin or axonal loss. ECS significantly reduced demyelination (69% reduction, **E**) or axonal loss (97% reduction, **F**). Values are presented as percentage of the average area of demyelimation/axonal loss out of the total WM area. (**G**–**N**) Confirmation of histochemical analysis by immunofluorescence staining for myelin and axonal markers. Serial adjacent sections were stained in uninjured and lesioned WM of chronic EAE mice. In uninjured WM, Gold-Black staining (**G**) correlated well with strong myelin basic protein (MBP) staining (**H**), and Bielschowsky staining (**I**) with neurofilament M (NF) staining (**J**). In lesions, loss of myelin (as found by Gold-Black, **K**) correlated with marked reduction in MBP (**L**), and loss of axons (as found by Bielschowsky, **M**) with reduced NF staining (**N**). (**O**–**Q**) Immunofluorescence staining for APC in control EAE (**O**) and ECS-treated EAE (**P**) showed no difference in APC^+^ cells in SC WM (**Q**). APC^+^ cell quantification is provided as number of cells per microscopic field. (**R**–**T**) Immunofluorescence staining for NG2 in control EAE (**R**) and ECS-treated EAE (**S**) showed a 57% reduction in NG2^+^ cells in SC WM (**Q**)**.** NG2^+^ cell quantification is provided as the number of cells per microscopic field. *P* values calculated with Student’s unpaired *t* test. Box-and-whisker plots show quartiles with median, and with minima and maxima at the bottom and top whiskers, respectively.

The beneficial clinical effect of ECS on the acute relapse prompted us to examine the value of repeated ECS treatments on the course of chronic EAE. We therefore examined the effect of ECS treatment, delivered initially intensely in daily sessions, followed by long-term weekly ECS maintenance. To address the clinical situation of chronic disease, Biozzi EAE mice were subject to ECS sessions starting after the acute relapse, just before onset of the progressive phase. Three consecutive ECS sessions were started on days 35–37 p.i. (*n* = 17) and were continued by weekly maintenance treatments. Two control groups were used: a sham treatment group (control, *n* = 16) and a group receiving a subthreshold electrical current of 0.4 mC (*n* = 8), in order to account for any potential effects of an electric shock that does not stimulate distal nervous tissue, and in particular the spinal cord, where the neuroinflammatory process is most abundant. Marked and long-lasting clinical improvement was evident in mice treated by ECS (Figure 1C). These mice were less likely to relapse (Figure 1D), and their overall burden of disease was milder, as represented by a lower cumulative score during the chronic phase (days 35–95 p.i.) (Figure 1E). Mice treated with a subthreshold electric dose relapsed at the same rate as sham-treated mice, although this occurred several days later (probably due to the mere stress associated with the electric current). However, this group “caught up” with the sham-treatment group, and there was no difference in its mean cumulative score. Pathological analyses was performed after the long-term weekly maintenance ECS therapy (at the end of the experiment) on day 95 p.i., as shown in Figure 1C. Immunofluorescence staining showed that ECS-treated EAE mice exhibited significantly less T cell infiltration (59% reduction, *P* = 0.03; Figure 2, A–E) and fewer IBA1^+^ activated microglia/macrophages (44% reduction, *P* = 0.02; Figure 2, F–H) in the WM of the SC, as compared with control EAE mice. In addition, GFAP staining indicated a borderline significant (*P* = 0.054) 35% reduction in astrogliosis in ECS-treated EAE mice as compared with control EAE mice (Figure 2, I–K). As neuroinflammation drives the demyelinating process and its associated axonal injury, we examined whether attenuation of neuroinflammation by ECS resulted in neuroprotection. Indeed, ECS treatment resulted in significantly less demyelination in the SC and nearly no axonal loss, as compared with sham-treated EAE mice (Figure 3, A–N). We further quantified and compared the number of APC^+^ oligodendrocytes and NG2^+^ oligodendrocyte-progenitor cells. There was no difference in the number of oligodendrocytes between experimental groups (Figure 3, O–Q). There was an increase in NG2^+^ cells in control EAE spinal cords (3.8-fold increase in relation to naive spinal cord), possibly reflecting a proliferative response to the demyelinating process (Figure 3, R and T). In ECS-treated EAE mice, there was just marginal increase in NG2^+^ cells (Figure 3, S and T), in agreement with the marked attenuation of neuroinflammation and of demyelination. Thus, ECS attenuates chronic neuroinflammation in progressive EAE and prevents inflammation-induced demyelination and axonal loss in a clinically relevant model of chronic MS.

### ECS attenuates neuroinflammation by direct targeting of the CNS.

**Figure 4:**
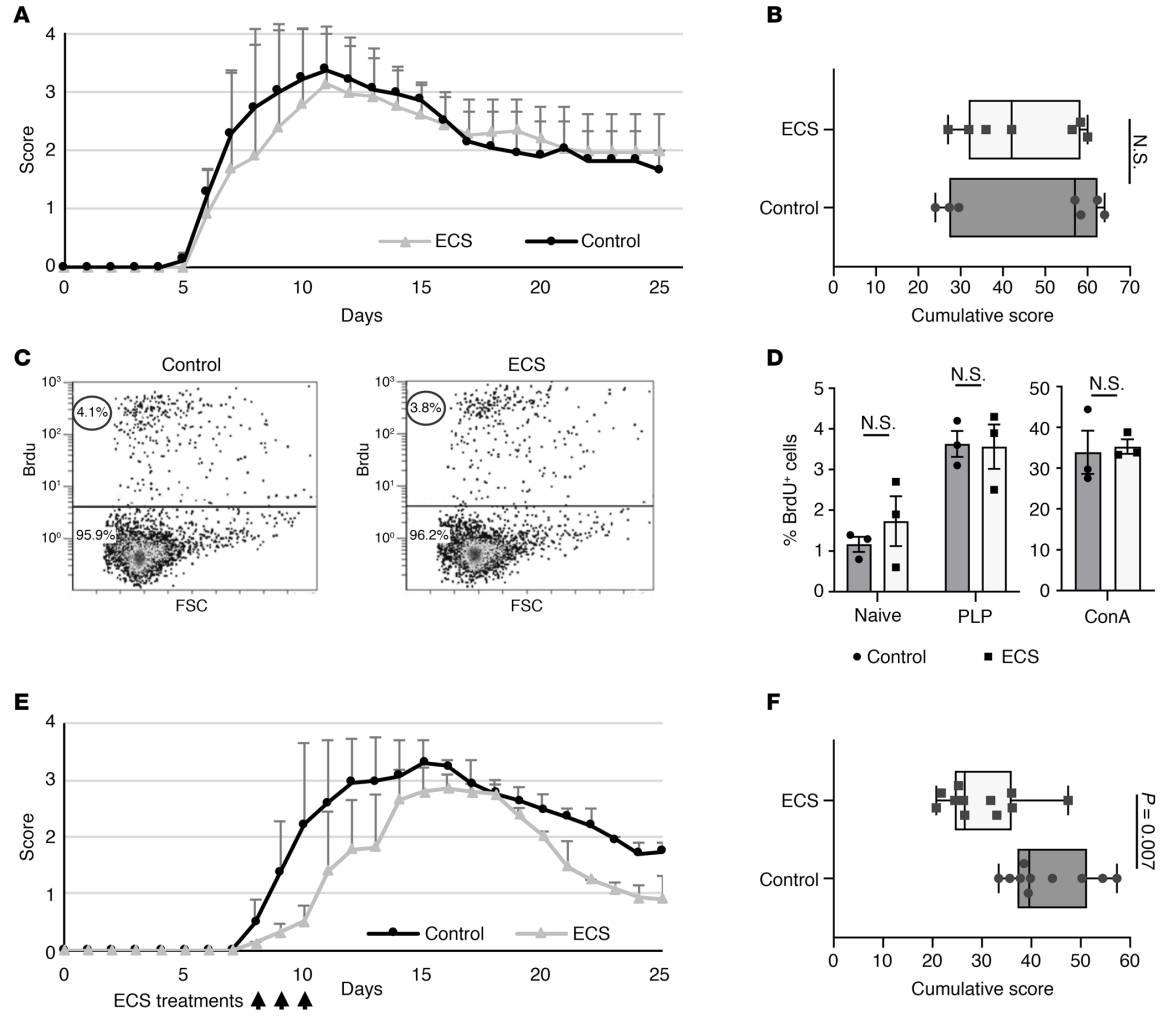
ECS attenuates EAE by a direct effect on the CNS. EAE was induced in SJL mice by transfer of encephalitogenic T cells from donor mice to recipient mice. (**A** and **B**) Donor mice received 3 consecutive ECS (*n* = 7) or sham (control, *n* = 7) treatments on days 8–10 p.i. Twenty-four hours later, lymph node cells were isolated from ECS-treated or the control group and injected into 2 groups of naive mice (day 0). Disease severity and pattern were similar between the 2 recipient groups. Data represent mean ± SD (**A**). No significant difference was found in the cumulative score between the mice that received encephalitogenic lymphocytes from the control or the ECS-treated group (**B**). The experiment was repeated twice. (**C** and **D**) Encephalitogenic lymphocytes from control or ECS-treated mice were activated in vitro with PLP or ConA. Representative FACS image (from 3 independent experiments) of Brdu^+^ cells showing similar percentage of proliferating cells from ECS and sham-treated donor mice in response to PLP (**C**). FACS analysis of Brdu-stained cells showed no significant difference in the percentage of Brdu^+^ cells between the groups when not activated (naive) or activated with PLP (quantification of **C**) or ConA (**D**). Three independent experiments were performed. Data represent mean ± SEM. (**E** and **F**) Recipient mice treated with 3 consecutive ECS (*n* = 10 mice) or sham (*n* = 11 mice) treatments, starting at day 8 after transfer, upon first presentation of clinical signs. Disease severity and length was reduced among the ECS-treated group. Data represent mean ± SD (**E**). Cumulative score was significantly reduced in the recipient mice treated with ECS (**F**). The experiment was repeated twice. Box-and-whisker plots show quartiles with median, and with minima and maxima at the bottom and top whiskers, respectively. *P* values calculated with Student’s unpaired *t* test.

To investigate whether the therapeutic effect of ECS was mediated systemically via the peripheral immune system or directly on the CNS, we used the transfer EAE model in SJL/J mice. In this model, encephalitogenic autoreactive T cells are isolated from lymph nodes of proteolipid protein–immunized (PLP-immunized) mice (e.g., donor mice) and injected to recipient naive mice, which develop acute EAE. ECS therapy in donor mice may provide an indication as to its effect on peripheral immune responses, whereas ECS in recipient mice may provide an indication as to its direct effects on the CNS. Here, ECS sessions in PLP-immunized donor mice before lymph node cell (LNC) collection did not have any effect on their encephalitogenicity, resulting in similar onset and severity of EAE in recipient mice (Figure 4, A and B). Furthermore, LNCs from ECS-treated and from sham-treated immunized mice exhibited similar in vitro proliferative responses to the PLP-peptide autoantigen and to Concanavalin A (ConA; Figure 4, C and D). This suggests that ECS effect is not mediated via the systemic adaptive autoimmune response, although we cannot rule out the possibility that incubation of LNCs from ECS-treated donor mice with the autoantigen (or ConA) may have reversed a difference in the reactivity of T cells from the 2 experimental groups. When 3 consecutive daily ECS sessions were performed in recipient mice, starting at day 8 after transfer (first day of clinical signs, when encephalitogenic T cells have entered the CNS), there was significant attenuation of EAE (Figure 4, E and F). Thus, ECS probably does not induce its beneficial effect via the systemic immune system and attenuates neuroinflammation by direct targeting of the CNS.

**Figure 5:**
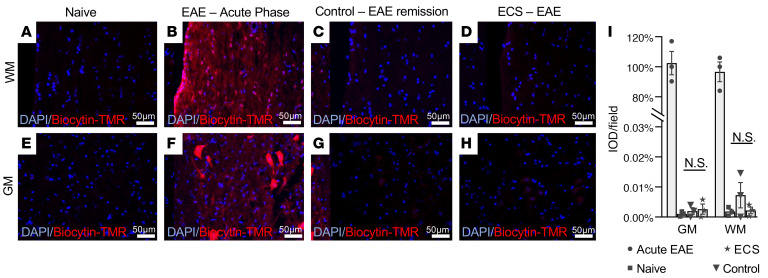
ECS does not affect the BBB permeability. Fluorescence microscopy in WM (**A**–**D**) and gray matter (GM, **E**–**H**) showed no Biocytin-TMR in the spinal cord of naive Biozzi ABH mice (*n* = 3 mice, **A** and **E**), robust fluorescence in Biozzi ABH mice during acute EAE relapse (*n* = 3 mice, **B** and **F**), and negligible fluorescence in sham-treated (control, *n* = 3 mice, **C** and **G**) and ECS-treated (*n* = 3 mice, **D** and **H**) spinal cords at day 38 p.i. (EAE remission), following 3–consecutive day treatment sessions. Biocytin-TMR fluorescence was measured as intensity optical density (IOD) and presented as percentage of fluorescence during peak of acute relapse (**I**). Data represent mean ± SEM.

We next examined the possibility that an ECS inhibitory effect on neuroinflammation was mediated by altering the BBB. BBB permeability (evaluated by using the Biocytin-TMR tracer) was compared between normal controls, untreated Biozzi EAE mice at the peak of acute (first) relapse, and ECS- and sham-treated mice after the initial 3 consecutive daily treatment sessions (e.g., day 38 p.i.; Figure 1C). The tracer did not penetrate the CNS in naive mice (Figure 5, A, E, and I), whereas massive penetration was observed in EAE mice at the peak of acute relapse (Figure 5, B, F, and I). At day 38 p.i. (during remission), there was minimal penetrance of tracer (less than 0.02% of penetrance during peak of acute relapse), which did not differ between ECS- and sham-treated mice (Figure 5, C, D, and G–I). Thus, ECS effect is mediated directly on the CNS, without any significant change in BBB permeability.

### ECS modulates microglial immune response and neurotoxicity.

**Figure 6:**
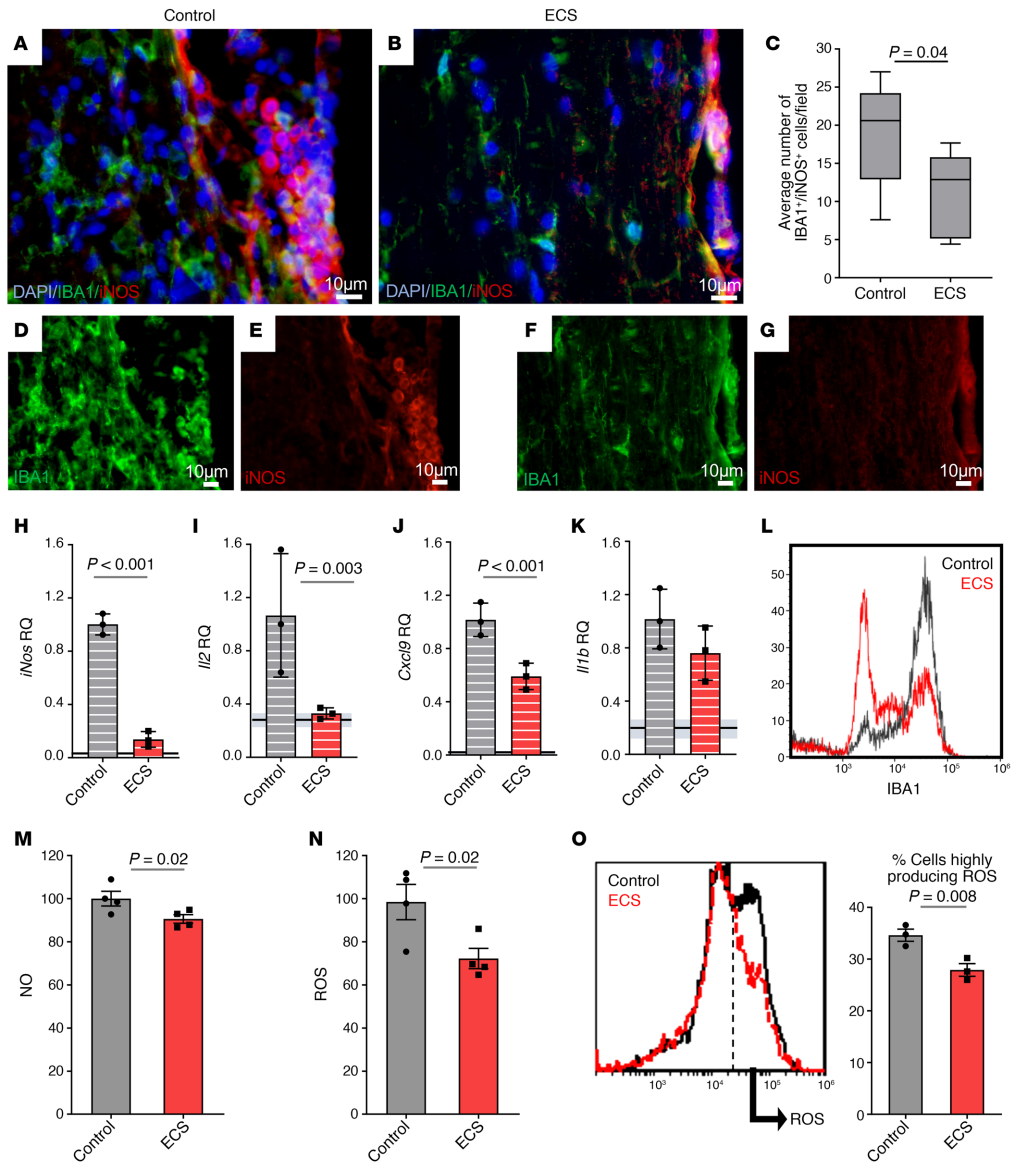
ECS decreases microglial toxicity. (**A**–**G**) Immunofluorescence staining for IBA1 and iNOS in SC sections of sham-treated (control) (**A**) and ECS-treated (**B**) Biozzi ABH EAE mice at day 95 p.i. ECS significantly reduced the number of IBA1^+^iNOS^+^ cells in SC WM by 40%. Box-and-whisker plot shows quartiles with median, and with minima and maxima at the bottom and top whiskers, respectively (**C**). (**D**–**G**) Single-color panels of IBA1 and iNOS are shown for **A** (**D** and **E**, respectively) and for **B** (**F** and **G**, respectively). (**H**–**O**) CD11b^+^ cells were isolated at day 38 p.i., 24 hours after the last of 3 ECS treatments (performed on days 35–37 p.i.) and evaluated by RT-PCR, FACS analysis, and ELISAs. Experiments were repeated 3 or 4 independent times. (**H**–**K**) mRNA levels of *iNos* (**H**), *Il2* (**I**), *Cxcl9* (**J**) and *Il1b* (**K**) were reduced in CD11b^+^ cells isolated from ECS-treated as compared with sham-treated (control) Biozzi EAE mice. In all graphs, the horizontal lines (and gray zone) represent the RQ mean value (±RQ min/max) of naive, age-matched Biozzi mice. (**L**) FACS analysis of IBA1 expression showed marked reduction in IBA1 expression (and specifically in IBA1^hi^ cells) in CD11b^+^ cells, isolated from ECS-treated spinal cords, as compared with sham-treated (control) mice (representative image). (**M**–**O**) Levels of NO (**M**) and ROS (**N**) production were decreased in isolated CD11b^+^ cells from ECS-treated as compared with control EAE mice, measured by ELISA reader in response to Griess Reagent or DCFDA, respectively. FACS analysis (**O**) of intracellular ROS (response to DCFDA) in the isolated CD11b^+^ cells from EAE mice showed 2 cell populations: low and high ROS–producing cells (representative image). ECS reduced the percentage of high ROS–producing CD11b^+^ cells. *P* value calculated with Student’s unpaired *t* test. Error bars: RQ min/max in **H**–**K** and SEM in **M**–**O**.

**Figure 7:**
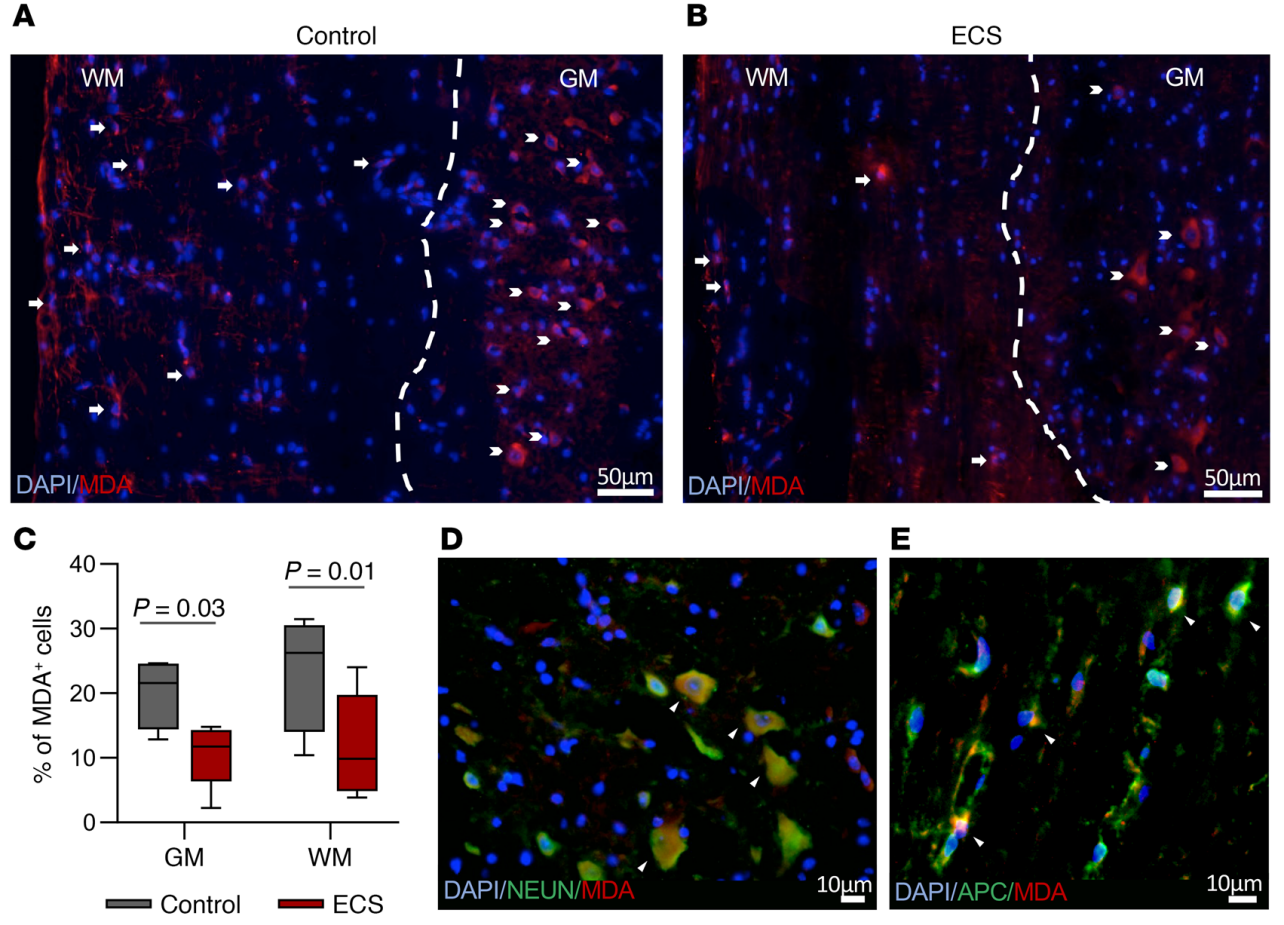
ECS reduces oxidative injury in spinal cords of ECS-treated EAE mice. (**A** and **B**) Immunofluorescence staining for MDA in SC sections of EAE Biozzi mice (day 95 p.i.). ECS reduced the number of oxidative injured MDA^+^ cells (shown as fraction of total cells) within the white matter (WM) and gray matter (GM) of the SC (**C**). In **A** and **B**, the dashed lines demarcate the WM/GM border, arrows point at MDA^+^ cells in the WM, and chevron arrows point at MDA^+^ cells in the GM. (**D**) Double staining for NeuN (marker for neurons) and MDA showing localization of MDA staining in neurons in the GM of EAE mice, marked by arrowheads. (**E**) Double staining for APC (marker for oligodendrocytes) and MDA showing localization of MDA staining in oligodendrocytes in the WM of EAE mice, marked by arrowheads. Box-and-whisker plot shows quartiles with median, and with minima and maxima at the bottom and top whiskers, respectively. *P* values calculated with Student’s unpaired *t* test.

In progressive MS, activated neurotoxic microglia play a central role in the pathogenesis of disease. Since ECS inhibited chronic EAE, affecting mostly the spinal cord, we hypothesized that electric stimulation of the cortex by ECS may be transmitted along the neuroaxis to modulate neuroinflammation in distal spinal tracts. Furthermore, since ECS targeted the CNS directly, without affecting peripheral T cell encephalitogenicity, we sought to examine its effect on the spinal cord innate immune system. Inducible NOS–positive (iNOS^+^) microglia are known to mediate neurotoxicity in chronic neuroinflammatory and neurodegenerative disorders, with NO and ROS playing a central role in microglial neurotoxicity (2). In chronic EAE mice (day 95 p.i.), there were significantly fewer iNOS^+^IBA1^+^ cells in spinal WM tracts of ECS-treated mice as compared with sham-treated mice (Figure 6, A–G). To further characterize the effect of ECS on the innate immune response, we isolated CD11b^+^ cells from spinal cords of ECS- or sham-treated Biozzi EAE mice 1 day after the 3-day ECS treatment initiation (day 38 p.i.). The expression of *iNos*, proinflammatory, and antiinflammatory cytokines and of chemokines was examined by reverse transcription PCR (RT-PCR). ECS induced a 90% inhibition (*P* < 0.001) of the highly expressed *iNos* mRNA in EAE mice (Figure 6H) and reduced the expression of *Il2* (Δrelative quantification [RQ] = –0.67, *P* = 0.003) and *Cxcl9* (ΔRQ = –0.41, *P* = 0.001), which are involved in stimulating and attracting T cells (Figure 6, I and J). These may explain the reduced T cell infiltration to the CNS in ECS-treated mice. In comparison with EAE mice, in ECS-treated EAE mice there was a trend for inhibition of *Il1b* (Figure 6K) and a nonsignificant effect on *Cxcl10* (ΔRQ = 0.07, *P* = 0.3), *Ccl2* (ΔRQ = –0.2, *P* = 0.2), *Ccl3* (ΔRQ = 0.3, *P* = 0.07), *Ccl4* (ΔRQ = 0.48, *P* = 0.1), *Il4* (ΔRQ = –0.35, *P* = 0.3), *Il10* (ΔRQ = –0.002, *P* = 0.5), and *Tgfb* (ΔRQ = –0.014, *P* = 0.5). Of note, ECS treatment reduced *iNos*, *Cxcl9*, and *Il1b* expression, albeit not to the levels found in naive animals (Figure 6, I–K). We then performed ex vivo functional assays on freshly isolated CD11b^+^ cells from spinal cords of sham- and ECS-treated EAE mice on day 38 p.i. and examined, by FACS analysis, the level of IBA1 expression as an activation marker. In ECS-treated EAE mice, there was marked inhibition in the level of IBA1 expression (Figure 6L), with an increase in the population of IBA1^lo^ on the expense of IBA1^hi^. Furthermore, CD11b^+^ cells isolated from ECS-treated EAE mice produced significantly less NO and ROS (Figure 6, M and N) than cells isolated from sham-treated EAE mice. FACS analysis indicated the existence of high and low ROS–producing CD11b^+^ cell populations in EAE mice. ECS reduced the fraction of high ROS–producing cells (Figure 6O). These results collectively suggest that ECS prevents a neurotoxic phenotype of microglia without affecting their basic immune functions, such as cytokine response. To further examine whether ECS prevents a neurotoxic phenotype of microglia, we evaluated the oxidative stress in EAE mice. Formation of ROS causes degradation of lipids, resulting in malondialdehyde (MDA) production, which can therefore serve as an indicator of oxidative injury. To examine whether the reduction in iNOS^+^, ROS-producing microglia was associated with a reduction in oxidative stress, we stained for MDA in the spinal cords of EAE mice at the end of clinical follow-up (day 95 p.i.). In ECS-treated mice, there was a significant decrease in MDA^+^ cells both in the white and gray matter of the spinal cords (Figure 7, A–E). Thus, ECS reduces the neurotoxic phenotype of microglia/macrophages in EAE, as shown by the expression of neurotoxic markers, production of oxidants in functional assays, and the pathologic outcome of tissue oxidative injury.

## Discussion

We examined here the effects of brain stimulation by ECS on chronic active neuroinflammation using the EAE model in Biozzi mice, a model that is reminiscent of chronic MS. Our study provides the first demonstration to our knowledge that ECS inhibits neuroinflammation clinically and pathologically and that it protects from inflammation-driven neurodegeneration, reducing the extent of demyelination and axonal loss. Importantly, ECS was effective in a clinically relevant disease model, when administered after disease onset in an established disease, using a standard clinical protocol in terms of electrical dose and frequency of treatments. Furthermore, repeated ECS sessions produced an ongoing therapeutic effect that is crucial for chronic-active brain disorders. These observations make this mode of therapy and its mechanism of action a valid therapeutic target for clinical translation.

ECS attenuated major features of chronic neuroinflammation, as evident by reductions in T cell infiltration, astrogliosis and microgliosis. The immunomodulatory effect of ECS was mediated in part by targeting of the CNS innate immune system and reducing microglial neurotoxicity. It is widely accepted that microglia affect neuronal function in health and disease (36) and that activated toxic microglia drive neurodegeneration in chronic neuroinflammatory and neurodegenerative diseases (11, 37). Our study provides a link between electrical activity and microglial function. We used here suprathreshold electric stimulation to transmit the therapeutic effect from the cortex to the spinal cord and showed that electric neurostimulation can drive microglial health and reverse their hyperactive neurotoxic phenotype to attenuate neuroinflammation. This is supported by recent studies that describe immunomodulatory effects in the spinal cord by transcutaneous electrical nerve stimulation (38) and in the brain by vagal nerve stimulation (39, 40). Indeed, several neuron-derived chemokines, cytokines, and neurotransmitters have been shown to regulate microglial functions (41, 42). Our findings are in agreement also with the wider concept of neural activity–dependent restoration of brain health, which is supported by observations of promotion of rewiring (43) and of oligodendrogenesis and myelination (44) by neuronal activity.

Current MS therapies target the peripheral adaptive immune system, modifying the relapsing-remitting phase of the disease. They are not useful in progressive MS (13), where systemic autoimmunity declines, and the disease is driven in a compartmentalized manner from within the CNS (9). In progressive MS, microglia seem to lose their normal homeostatic phenotype (45) and are activated throughout the CNS (46), producing toxic chemokines, cytokines, NO, and ROS. The direct targeting of neurotoxic microglia by ECS underscores the pivotal role of activated microglia in driving CNS injury in chronic active EAE and MS (9, 47). ECS did not prevent the formation of highly encephalitogenic T cells, but rather affected brain innate immune cells and prevented microglial and innate immunity-mediated neuroinflammation, demyelination, and axonal loss.

The implications of these findings address several neurodegenerative and neuropsychiatric diseases, in addition to MS. Clinically, ECT is the most effective treatment in depression and is the treatment of choice in drug-resistant depression (48). There is increasing evidence that activated microglia play an important role in the pathogenesis of depression (27, 49). Thus, our findings provide an attractive mechanism by which ECT may affect and reverse depression. Also, microglial neurotoxicity has been shown to drive the neurodegenerative process in transgenic models of familial Alzheimer’s disease (50). Importantly, dysfunctional microglia, such as that caused by TREM2 mutations also promote Alzheimer’s disease pathogenesis (51, 52). These underscore the notion that both overactivation and suppression to a malfunctioning state of microglia are deleterious to brain health and that restoration of microglial homeostasis is the preferred therapeutic target, rather than their elimination or total inhibition. Our findings suggest that ECS may reduce microglial toxicity, without eliminating their ability to produce immune mediators. Importantly, ECS did not cause a general suppressive effect on microglia, nor a significant effect on cytokines associated with an M1 versus M2 phenotype. Rather, ECS reversed the microglial neurotoxic phenotype, as indicated by reducing iNOS expression, reducing NO and ROS production, and reducing the expression of *Il2* and *Cxcl9* as T cell stimulating and attracting cytokines. The lack of a general suppressive effect of ECS on microglial function is important in enabling the still-activated, ECS-treated microglia to participate in their homeostatic roles (53), as well as in regenerative processes (54). While we show a remarkable inhibitory effect of ECS on neuroinflammation, this does not rule out additional mechanisms of action, affecting the resilience of the various populations of brain cells.

In conclusion, ECT induces an immunomodulatory therapeutic effect in a clinically relevant setting of experimental chronic MS. CNS microglia serve as key therapeutic targets for chronic-progressive MS, and modulation of their neurotoxicity by ECT may considerably reduce their neurotoxicity. These findings may bare implications to other neurodegenerative and neuropsychiatric diseases driven by microglial neurotoxicity, such as Alzheimer’s disease (50) and major depression (55).

## Methods

### EAE.

Biozzi ABH and SJL/J female mice (Envigo) were bred and grown under specific pathogen–free conditions. Animal experiments were approved by the IRB: MD-14-14089-4, MD-17-14988-5.

EAE was induced in 6- to 8-week-old Biozzi ABH mice using spinal cord homogenate (SCH) as described (33, 56) and were scored daily as follows: 0, asymptomatic; 1, loss of tail tonicity; 2, difficulty in rolling over; 3, hind leg weakness or limping; 4, hind leg paralysis; 5, full 4-leg paralysis.

For transfer EAE, 6- to 7-week-old female SJL/J mice were immunized with 175 μg PLP 139–151 peptide in 100 μL saline and equal volume of CFA, containing 5 mg/mL H37RA (Difco Laboratories). Ten days after immunization, LNCs were harvested. A total of 3 × 10^6^ cells/mL were cultured for 72 hours in the presence of 100 μg/mL PLP 139–151 peptide. A total of 15 × 10^6^ cells were injected i.p. into recipient 6- to 7-week-old female SJL/J mice. EAE signs were observed clinically around day 8 p.i. Mice were followed clinically up to day 25 p.i. and scored as previously described (57).

### ECS.

ECS was applied via ear clip electrodes using the Ugo Basile ECT unit (model 57800). ECS parameters were set on frequency, 100 Hz; pulse width, 0.5 ms; shock duration, 1 second. The electric threshold is the lowest dose causing a full seizure. Threshold was established by changing the current in intervals of 2 mA, starting at 8 mA. All ECS sessions were then given using a current of 24 mA equal to an electric dose of 2.4 mC (according to the equation: Q electric dose [milliCoulombs, mC] = I current [A] × PW pulse width [mS] × 2 times the frequency [Hz] × D duration [S]) (58). All control mice were sham treated by attaching ear clip electrodes without applying a current.

Three experimental ECS protocols were used. (a) ECS was applied individually on the first day of clinical signs in EAE Biozzi mice, followed by 4 ECS treatments on alternating days. (b) ECS (and an additional control group receiving subthreshold electric dose of 0.4 mC [4 mA current]) was applied to Biozzi mice starting on day 35 p.i. of EAE for 3 consecutive days (35–37 p.i.) followed by once-weekly ECS treatment up to day 95. (c) In the transfer EAE model, SJL/J donor mice were subject to 3 consecutive ECS sessions on days 7–9, and lymph nodes were harvested on day 10 for culture and transfer experiments. In other experiments, recipient mice received 3 consecutive ECS treatments on days 8–10 after LNC injection (with onset of EAE clinical signs).

All experiments were repeated 2–4 times; the number of repeats and the number of samples (*n*) in each experimental group are specified in figure legends.

### Histopathology.

Animals were anesthetized with pentobarbital and perfused with ice-cold PBS followed by cold 4% paraformaldehyde. Spinal cords were placed in OCT and deep frozen in dry ice. Serial 10 μm longitudinal sections of spinal cords were prepared, and immunofluorescence stainings were performed as previously described (59). The following antibodies were used: anti-CD3 (Bio-Rad Laboratories, MCA1477), anti-IBA1 (Fujifilm Wako, 019-19741), anti-iNOS (Novus Biologicals, NBP2-22119), anti-NEUN (MilliporeSigma, MAB377), anti-APC (Ab-7, Calbiochem, OP80), anti-MDA (Abcam, ab6463), anti-GFAP (Dako, Z0334), anti-NG2 (MilliporeSigma, AB5320), anti-MPB (MilliporeSigma, MAB386), and anti-NF M (Chemicon International, AB1987). Goat anti–rabbit Alexa Fluor 488 (Invitrogen, Thermo Fisher Scientific, A11034), goat anti–mouse Alexa Fluor 488 (Invitrogen, Thermo Fisher Scientific, A11001), goat anti–rabbit Alexa Fluor 555 (Invitrogen, Thermo Fisher Scientific, A27039), goat anti–rat Alexa Fluor 555 (Invitrogen, Thermo Fisher Scientific, A21434), and goat anti–mouse Alexa Fluor 555 (1:200, Invitrogen, Thermo Fisher Scientific, A28180) were used as secondary antibodies appropriately. Nuclear counterstain was performed using DAPI (Vector Laboratories). Bielschowsky silver impregnation (for axons) and Gold-Black (for myelin) staining were performed as previously described (60).

### Quantification of axonal and myelin loss.

Analysis was performed on longitudinal sections ventral to the central canal, in a consistent manner. Degree of loss was assessed by measuring the area of injury out of total WM area using ImageJ software (ver. 1.51H, NIH). Four sections, 60 μm apart, were quantified per spinal cord, and average percentage ± SEM of axonal or myelin area loss was calculated.

### Quantification of gliosis and inflammatory response.

CD3^+^ T cells were counted along the meninges and WM on both sides of the cervical spinal cord (cut uniformly in 7 mm longitudinal sections) and quantified as number of cells per section. IBA1^+^ cells, IBA1^+^iNOS^+^ cells, GFAP^+^ cells, APC^+^ cells, and NG2^+^ cells were counted manually in WM and associated meninges in 6 microscopic fields (×40 power of objective, ×10 power of eyepiece) for each section. Four sections, 60 μm apart, were quantified per mouse, followed by calculating group average cell number per microscopic field ± SEM. All experiments were conducted in a blinded manner, adhering to stereological principles.

### BBB permeability.

Biozzi ABH mice groups (3 mice per group) were evaluated: naive mice, EAE mice at peak of the first relapse (day 18 p.i.), and EAE mice at day 38 p.i. (remission) following 3 consecutive ECS or sham sessions. A total of 1 mg of 5-(and 6)-tetramethylrhodamine biocytin (Biocytin-TMR, Invitrogen, Thermo Fisher Scientific) diluted in 100 μL PBS was injected per mouse into the tail vein. Penetration into the blood circulation was indicated by pink colorization of the ears within seconds. Thirty minutes after injection, animals were anesthetized and perfused as described above. Spinal cords were extracted and deep frozen in dry ice, and serial 10 μm longitudinal sections were prepared. Nuclear counterstain was performed using DAPI (Vector Laboratories).

Images (×40 power of objective, ×10 power of eyepiece) from white and gray matter were captured using identical laser intensity, exposure times, and magnification in all cohorts. To set these parameters, livers from tracer-injected (positive control) and noninjected mice (negative control) were used. Fluorescence intensity analysis was performed. Eight images per section, from 4 sections 60 μm apart, were quantified (as intensity optical density; IOD) using Image-Pro Plus software (Media Cybernetics), and the group average IOD per microscopic field ± SEM was calculated. All experiments were conducted in a blinded manner, adhering to stereological principles.

### MDA quantification and analysis.

Designated fields in the spinal cord were scanned and captured (×40 power of objective, ×10 power of eyepiece) using the Nikon-TL Imaging microscopic system, and all DAPI^+^ cells in each image were counted along the WM and gray matter separately. A total of 150–250 cells per each WM or GM area were counted in each image. The fraction of the MDA^+^ cells out of the total cell count was calculated. Four images (of sections 60 μm apart) were quantified per mouse, followed by calculating group average ± SEM. All experiments were conducted in a blinded manner.

### LNC proliferation assay.

LNCs from ECS- or sham-treated SJL/J mice were isolated 10 days after PLP immunization and cultured for 72 hours with ConA (2.5 μg/mL, MilliporeSigma) or PLP 139–151 peptide (100 μg/mL). Proliferation was assessed using BrdU incorporation as previously described (61).

### Microglia/macrophage isolation and FACS analysis.

Spinal cord tissue from Biozzi ABH EAE mice was dissociated to single-cell suspension at day 38 p.i. following 3–consecutive day ECS or sham sessions, using the Neural Tissue Dissociation Kit (Miltenyi Biotec). Myelin was removed using Percoll (GE Healthcare) followed by microglia/macrophages isolation using CD11b microbeads and MS columns (Miltenyi Biotec) according to manufacturer instructions. Degree of microglia/macrophages enrichment was assessed by consequent CD11b (BD Biosciences, M1/70) staining and FACS analysis (Beckman Coulter); in all experiments at least 75% of isolated cells expressed CD11b. IBA1 staining was performed with anti-IBA1 antibody (Fujifilm Wako, 019-19741), following staining with secondary antibody (goat anti–rabbit Alexa Fluor 555, 1:100, Invitrogen, Thermo Fisher Scientific) using the fixation buffer and intracellular staining Perm Wash buffer (BioLegend), and analyzed by FACS (Beckman Coulter).

### NO and ROS measuring.

Spinal cord tissue from Biozzi ABH EAE mice were dissociated to single-cell suspension at day 38 p.i. following 3–consecutive day ECS or sham sessions, and microglia/macrophages were isolated using CD11b microbeads as described above. Freshly isolated microglia/macrophages were seeded on poly-L-lysine–covered 96-well plates. Cells were activated overnight with LPS (2 ng/mL, *E. coli* O111:B4, MilliporeSigma). NO production was assessed (on 250 × 10^5^ cells/well) using Greiss Reagent System according to manufacturer’s protocol (Promega, G2930) and quantified using ELISA plate reader (Tecan Spark 10M). ROS production was measured (on 50 × 10^5^ cells/well) using DCFDA dye according to manufacturer’s protocol (Abcam, ab113851) and quantified using ELISA plate reader (Beckman Coulter DTX 880 multimode detector). To measure intracellular ROS, freshly isolated microglia/macrophages were stained with DCFDA, and cell fluorescence was quantified using FACS.

### Real-time PCR.

RNA was isolated from microglia/monocytes using RNeasy Plus Mini Kit (QIAGEN). cDNA was generated from a concentration of 50 μg/mL RNA using qScript cDNA Synthesis Kit (Quanta Biosciences). Reaction mixture included 1 μL of cDNA, 300 nM of appropriate forward and reverse primers (Agentek), and 5 μL PerfeCTA SYBR Green FastMix ROX (Quanta Biosciences) to a total volume of 10 μL. Gene amplification was carried out using the StepOnePlus real-time PCR system (Applied Biosystems). Primers used:

*iNos* forward (F): 5′ CCCAGCCTTGCATCCTCAT 3′; *iNos* reverse (R): 5′ ATGCGGCCTCCTTTGAGC 3′.

*Il1b* F: 5′ CCTGAACTCAAGTGTGAAATGCC 3′; *Il1b* R: 5′ TCATCAGGACAGCCCAGGTC 3′.

*Il2* F: 5′ AGGATGGAGAATTACAGGAACCTG 3′; *Il2* R: 5′ CTTTCAATTCTGTGGCCTGCT 3′.

*Cxcl9* F: 5′ GCAGTGTGGAGTTCGAGGAA 3′; *Cxcl9* R: 5′ CTGTTTGAGGTCTTTGAGGGATTTG 3′.

*Cxcl10* F: 5′ GTCTGAGTGGGACTCAAGGGA 3′; *Cxcl10* R: 5′ CAAGCTTCCCTATGGCCCTCAT 3′.

*Ccl2* F: 5′ ACGTGTTGGCTCAGCCAGAT 3′; *Ccl2* R: 5′ CAGCCTACTCATTGGGATCATCT 3′.

*Ccl3* F: 5′ CCAAGTCTTCTCAGCGCCATAT 3′; *Ccl3* R: 5′ TGGAATCTTCCGGCTGTAGGA 3′.

*Ccl4* F: 5′ CGTGTCTGCCCTCTCTCTCC 3′; *Ccl4* R: 5′ GGAGGGTCAGAGCCCATTG 3′.

*Il4* F: 5′ CGTCCTCACAGCAACGAAGAA; *Il4* R: 5′ CACCTTGGAAGCCCTACAGAC.

*Il10* F: 5′ CAGCCAGGTGAAGACTTTCTTTC 3′; *Il10* R: 5′ CTGCATTAAGGAGTCGGTTAGCA 3′.

*Tgfb* F: 5′ CTGAACCAAGGAGACGGAATACA 3′; *Tgfb* R: 5′ GGGCTGATCCCGTTGATTT 3′.

### Statistics.

Statistical analysis was performed using SPSS, version 24. The statistical tests used in this manuscript were Student’s 1-tailed unpaired *t* test, 3-way χ^2^ test, or 1-way ANOVA followed by Tukey’s post hoc test, as appropriate. The test used was specified for each experiment in the figure legends. *P* < 0.05 was considered significant.

### Study approval.

Animal experiments were approved by the Hebrew University – Hadassah Medical Center IRB committee (MD-14-14089-4, MD-17-14988-5).

## Author contributions

SG contributed by designing research studies, conducting experiments, acquiring data, analyzing data, and writing the manuscript. NF contributed to designing research studies and conducting experiments. TBH contributed by designing research studies, analyzing data, and writing the manuscript.

## Version 1 08/11/2020

In-Press Preview

## Version 2 09/03/2020

Electronic publication
